# Characterization of a Chitosanase from Jelly Fig (*Ficus awkeotsang* Makino) Latex and Its Application in the Production of Water-Soluble Low Molecular Weight Chitosans

**DOI:** 10.1371/journal.pone.0150490

**Published:** 2016-03-03

**Authors:** Chen-Tien Chang, Yen-Lu Lin, Shu-Wei Lu, Chun-Wei Huang, Yu-Ting Wang, Yun-Chin Chung

**Affiliations:** Department of Food and Nutrition, Providence University, Taichung, Republic of China (Taiwan); Michigan Technological University, UNITED STATES

## Abstract

A chitosanase was purified from jelly fig latex by ammonium sulfate fractionation (50–80% saturation) and three successive column chromatography steps. The purified enzyme was almost homogeneous, as determined by SDS-polyacrylamide gel electrophoresis (SDS-PAGE) and gel activity staining. The molecular mass of the enzyme was 20.5 kDa. The isoelectric point (p*I*) was <3.5, as estimated by isoelectric focusing electrophoresis on PhastGel IEF 3-9. Using chitosan as the substrate, the optimal pH for the enzyme reaction was 4.5; the kinetic parameters *K*_m_ and *V*_max_ were 0.089 mg mL^-1^ and 0.69 μmol min^-1^ mg^-1^, respectively. The enzyme showed activity toward chitosan polymers which exhibited various degrees of deacetylation (21–94%). The enzyme hydrolyzed 70–84% deacetylated chitosan polymers most effectively. Substrate specificity analysis indicated that the enzyme catalyzed the hydrolysis of chitin and chitosan polymers and their derivatives. The products of the hydrolysis of chitosan polymer derivatives, ethylene glycol (EG) chitosan, carboxymethyl (CM) chitosan and aminoethyl (AE) chitosan, were low molecular weight chitosans (LMWCs); these products were referred to as EG-LMWC, CM-LMWC and AE-LMWC, respectively. The average molecular weights of EG-LMWC, CM-LMWC and AE-LMWC were 11.2, 11.2 and 8.89 kDa, respectively. All of the LMWC products exhibited free radical scavenging activities toward ABTS^•+^, superoxide and peroxyl radicals.

## Introduction

Approximately 8.9% of angiosperm plants exude latex from a tubular structure called a laticifer when tissues are damaged [1]. Latex is composed of abundant chemicals and enzymes, such as terpenoids, alkaloids, rubber, cardenolides, β-1,3-glucanase, hevamine, hevein, chitosanase and β-N-acetylhexosaminidase [2–13]. Previous research has suggested that latex secretion is a defense against wounding and/or predators such as insects and microorganisms. The major defensive roles that plant latex plays include trapping and immobilizing herbivorous insects due to its sticky nature. Some chemical ingredients, such as alkaloids and cardenolides, are toxic to animals and insects. In addition, chitinase-related enzymes are able to hydrolyze fungal pathogens [14–18]. The defensive substances in plant latex are induced by mechanical damage and damage by herbivores [19–22].

Chitinases are commonly found in plant latex, and their defensive mechanisms against fungi are well known [23–27]. Chitosanase are widely present in microorganisms; however, few chitosanases have been found in plants and animals [28]. There have been no reports on the presence of chitosanase in latex, except for ficin [29]. The rarity of chitosanases in latex obscures their nature and applications.

Chitosanase (EC 3.2.1.132) hydrolyzes chitosan to generate low molecular weight chitosan and chitosan oligomers. Several studies have demonstrated that chitosan, a product of deacetylated chitin, has many special properties. These include improved antitumor activity, superior antioxidant activity, immune system stimulation and cholesterol reduction [30,31]. However, high molecular weight chitosans (HMWCs) have limited the commercial application. Depolymerized chitosan products, such as low molecular weight chitosan and chitooligosaccharides, can overcome these limitations; these products also have increased antitumor activity and can be an effective treatment for renal failure [12, 32,33,34,35].

Jelly fig (*Ficus awkeotsang* Makino), a cultivar exclusive to Taiwan, is a woody vine that grows in tropical and subtropical regions of the country [13]. The aqueous extracts of jelly fig achenes have been used to prepare jelly curd, which is an ingredient used in a summer drink popular in local markets. Ding et al. (2002) and Li et al. (2003) purified an acidic pectin methylesterase with a molecular mass of 38 kDa and a 30 kDa chitinase, respectively, from jelly curd prepared from jelly fig achene [36, 37]. Afterward, Chua et al. (2007) purified two thaumatin-like protein isoforms from jelly curd. However, related enzymes/proteins have not been identified in jelly fig latex [38].

Recently, we found that the latex exuded from the harvest jelly fig fruits contained high exo-glycosidic, proteolytic and chitinolytic activities. In this study, we found abundant proteins exhibiting various enzymatic activities in jelly fig latex. A chitosanase was further purified, and its characteristics were revealed. In addition, the jelly latex chitosanase was used to hydrolyze chitosan derivatives to produce low molecular weight chitosans (LMWCs). The antioxidant activities of these LMWCs are also reported.

## Materials and Methods

### Chemicals

Glucosamine, N-acetyl-D-glucosamine, neocuproine hydrochloride, nitroblue tetrazolium (NBT), phenazinemethosulfate (PMS), 2-chloroethanol, sodium chloroacetate, 2-chloroethylamine hydrochloride, *p*-nitrophenyl-α-D-glucopyranoside (*p*Np-α-Glc), *p*-nitrophenyl-α-D-galactopyranoside (*p*Np-α-Gal), *p*-nitrophenyl-β-D-glucopyranoside (*p*Np-β-Glc), *p*-nitrophenyl-β-D-galactopyranoside (*p*Np-β-Gal), *p*-nitrophenyl-β-D-xylopyranoside (*p*Np-β-Xyl), *p*-nitrophenyl-β-L-fucopyranoside (*p*Np-L-Fuc), *p*-nitrophenyl-N-acetyl-β-D-glucosaminide (*p*Np-β-GlcNAc), *p*-aminophenylmercuric acetate (*p*APMA), N-ethyl-N’-(3-dimethyl aminopropyl) carbodiimide (EDC), 2’2’-azobis (2-amidinitropropanoe) dihydrochloride (AAPH), 2’2-azino-bis-(3-ethylbenzthiazoline-6-sulfonic acid) diammonium salt (ABTS), 6-hydroxy-2,5,7,8-teramethylchroman-2-carboxylic acid (Trolox) and 3-methyl-2-bezothiazolene hydrazine hydrochloride (MBTH) were purchased from Sigma-Aldrich Fine Chemicals, Inc. (St. Louis, MO, USA). Dextran standards (58.6, 23.8, 5.22 and 1.27 kDa) were obtained from Fluka (Milwaukee, WI, USA). A bicinchoninic acid protein assay reagent was obtained from Pierce (Rockford, IL, USA). Chitooligosaccharides (COS, MW<5 kDa) was obtained from Roterm Trading Co., LTD. (Taiwan). Sephacryl S-100 HR column, Superose 12 HR 10/30 column, SDS-polyacrylamide gel electrophoresis LMW calibration kit, PhastGel IEF 3–9 and p*I* calibration kit (p*I* 3.5–9.3) were obtained from Pharmacia (Uppsala, Sweden).

### Preparation of a crude enzyme extract from jelly fig latex

Fresh latex collected from the fruits of a native specimen of jelly fig (*Ficus awkeotsang* Makino) grown in Fu-Chen farm, Taichung, Taiwan (http://www.goldfarm.idv.tw/html/index.asp). Fu-Chen farm is a private farm growing tropical and subtropical fruits. Mr. Yi-Fang Tein, the owner of the Fu-Chen farm, truly supported this study. No specific permission was required for growing jelly fig. Jelly fig was not on endangered or protected species lists in Taiwan. Upon arrival in the laboratory, the latex was dried by lyophilization and ground into powder. The lyophilized latex powder was stored at -20°C.

Five hundred milligrams of lyophilized jelly fig latex was dissolved in 50 mL of 25 mM imidazole-HCl buffer containing 1% polyvinyl pyrrolidone polymer (PVPP) at pH 7.4. The mixture was stirred with magnetic stirrer in a cold room for 1 h. Any insoluble substances were removed by centrifugation (15,000 *x g*, 30 min), and the resulting supernatant was further clarified by filtration through filter paper (Whatman no. 1) and microfiltration through a 0.45 μm membrane. The resulting filtrate was designated as the crude enzyme extract.

### Purification of chitosanase

The crude enzyme extract from jelly fig latex was fractionated by the addition of (NH_4_)_2_SO_4_. The precipitate formed between 50% and 80% (NH_4_)_2_SO_4_ saturation and was collected by centrifugation (15,000 *x g*, 30 min). The precipitate was then dissolved in 5 mL of 25 mM imidazole-HCl buffer (pH 7.4). After centrifugation at 15,000 *x g*, 30 min, the resulting supernatant was applied to a Sephacryl S-100 HR column (2.6 x 70 cm) and eluted with 25 mM imidazole-HCl buffer (pH 7.4) at a flow rate of 30 mL/h. Three and half-milliliter fractions were collected and assayed for chitosanase activity.

Fractions containing chitosanase were pooled and concentrated to approximately 5 mL via ultrafiltration with a 5-kDa molecular weight cutoff (MWCO) membrane (Millipore Co., Cork, Ireland). The concentrated fractions were applied to a *p*APMA-Sepharose 4B column (1.0 x 20 cm), which was prepared for affinity adsorption of cysteine protease as described previously [12]. After the sample was absorbed, the column was washed with 25 mM sodium phosphate buffer containing 0.5 M NaCl (pH 7.2) at a flow rate of 30 mL/h to elute the non-bound chitosanase. To elute the bound proteases, 0.15 M 2-mercaptoethanol in 25 mM sodium phosphate buffer was applied. Each fraction (4 mL) was monitored for absorbance at 280 nm. Fractions with demonstrated chitosanase and protease activities were collected.

The chitosanase obtained from the above affinity column was dialyzed against 25 mM imidazole-HCl (pH 7.4) and concentrated to approximately 4 mL by ultrafiltration as described above. The concentrated sample was then applied to a DEAE-Sephacel column (1.0 x 20 cm) that was pre-equilibrated with 25 mM imidazole-HCl (pH 7.4). After the sample was absorbed, the column was washed with equilibrium buffer until no protein was detected in the eluent. The bound chitosanase was eluted from the column with a linear NaCl gradient (0–0.5 M) in equilibrium buffer at a flow rate of 30 mL/h. Each fraction (3 mL) was monitored for absorbance at 280 nm and chitosanase activity. Fractions containing chitosanase were pooled.

### Preparation of chitin and chitosan derivatives

Glycol chitin (EG-chitin) or colloidal chitin was prepared according to methods described by Yamada and Imoto and Hirano and Naga, respectively. Glycol chitosan (EG-chitosan), carboxymethyl chitosan (CM-chitosan) and aminoethyl chitosan (AE-chitosan) were prepared according to the methods of Yamada and Imoto, Hirano and Glifford and Naoyuki, respectively [39–42].

### Measurement of chitosanase and chitinase activities

A 0.2 mL mixture containing 0.175 mL of 0.5% chitosan or chitinin in 0.1 M sodium acetate buffer (pH 4.5) and 0.025 mL of an appropriate dilution of enzyme was incubated at 50°C for 30 min. The reducing sugar produced was measured colorimetrically using neocuproine reagent as described by Dygert et al. [43]. One unit of chitosanase was defined as the amount of enzyme that produced 1 μmol of D-glucosamine per minute. One unit of chitinase was defined as the amount of enzyme that produced 1 μmol of N-acetyl-D-glucosamine per minute.

### Measurement of protease activity

Protease activity was measured using casein as a substrate according to the method of Anson, with some modifications. One protease unit was defined as the amount of enzyme that produced 1 μg of tyrosine per minute [44,45].

### Measurement of exo-glycosidase activity

The activities of exo-glycosidases, including α-glucosidase, β-glucosidase, α-galactosidase, β-galactosidase, β-xylosidase, β-L-fucosidase and β-N-acetylglucoaminidase, were determined by measuring the liberation of *p*-nitrophenol (as the phenolate anion) from the respective glycosides [12, 46]. One unit of exo-glycosidase was defined as the amount of enzyme that liberated 1 μmol of *p*-nitrophenol per minute.

### Sodium dodecyl sulfate-polyacrylamide gel electrophoresis (SDS-PAGE) and zymogram analysis

Samples were heated at 90°C for 5 min in SDS-PAGE sample buffer containing 2.5% (W/V) SDS and 5% 2-mercapotoethanol. SDS-PAGE was carried out in 12.5% polyacrylamide gels. For zymogram analysis, samples were incubated at room temperature for 2 h in SDS-PAGE sample buffer containing 2.5% (W/V) SDS and 15% sucrose without 2-mercaptoethanol. Zymogram analysis was performed according to Ouakfaoui and Asselinusing; 12.5% polyacrylamide gels containing 0.16% (W/V) glycol chitosan or glycol chitin were used, with bromophenol blue as the tracking dye [47,48,49]. After electrophoresis, the proteins were stained with Coomassie Brilliant Blue R-250 (CBR). Chitosanase or chitinase activity was detected using Calcofuor White M2R staining.

### Isoelectric focusing electrophoresis (IEF)

IEF was performed to determine the p*I* value of the purified chitosanase using a PhastGel IEF 3–9 apparatus. Carrier ampholytes were pre-focused at 75 Vh. The sample was focused at 410 Vh at 2.5 mA and 15°C. An 8 x 1 μL comb was used for sample loading. Following electrophoresis, the gels were stained with CBR.

### Determination of optimal pH and optimal temperature

The effects of pH on chitosanase activity were determined using chitosan as the substrate at 50°C as previously described; however, that enzyme was used in universal buffers with a pH range of 2.0–5.0 (Britton and Robinson type)[50]. The effects of temperatures ranging from 30 to 80°C on enzyme activity were determined at pH 4.5; chitosan was used as the substrate.

### Substrate specificity of chitosanase

The substrate specificity of the purified chitosanase was determined using natural and chemically modified chitin and chitosan as substrates under standard assay conditions. The amount of reducing sugar released was quantified colorimetrically as described for the standard assay.

### Determination of kinetic parameters

The initial reaction rates of purified chitosanase toward chitosan at different concentrations (0.044 to 0.44 mg mL^-1^) were determined at 50°C. The kinetic parameters *K*_m_ and *V*_max_ were calculated using Lineweaver-Burk plots.

### Hydrolysis of chitosan derivatives

Three hundred milligrams of lyophilized jelly fig latex were stirred in 50 mL of H_2_O with magnetic stirrer in cold room for 1 h. Any insoluble substances were removed by centrifugation (15,000 *x g*, 30 min). The resulting supernatant was incubated with 100 mL of 0.5% EG-chitosan, CM-chitosan or AE-chitosan in H_2_O for 24 h at 37°C. The reaction was stopped by heating the mixture in a 100°C water bath for 10 min. After centrifugation (10,000 *x g*, 10 min), the supernatant was stored at 4°C. The average molecular weights of the hydrolysates were measured in solution. The supernatant was lyophilized to determine the antioxidant activities of the hydrolysates.

### Determination of average molecular weights of chitosan hydrolysates

Chitosan hydrolysates were loaded onto a Superose 12 HR column (1.0 × 30 cm) and eluted with 0.1 M sodium acetate buffer (pH 4.5) at a flow rate of 30 mL/h. Each 0.5 mL fraction was collected and used for hexosamine determination [51].

### Measurement of ABTS radical-scavenging activity

The effects of LMWC derivatives and trolox on ABTS radicals were estimated according to the method of Re *et al*. [52]. An aliquot of LMWC derivatives (10 μL, 0.8–8 mg/mL) or trolox (0.05–1.0 mg/mL) was mixed with 990 μL of ABTS^․┿^ radicals. The mixture was left to stand for 10 min at room temperature in the dark. The absorbance of the resulting solution was measured spectrophotometrically at 734 nm. The capability to scavenge ABTS^․┿^ radicals was calculated using the following equation:

$$\text{scavenging}\;\text{effect}\;\left(\%\right)=\left[1-{\text{A}}_{\text{sample}}/{\text{A}}_{\text{control}}\right]\times 100\%$$

### Measurement of superoxide radical-scavenging activity

The effects of LMWC derivatives and trolox on superoxide radicals were determined using a PMS-NADH superoxide generating system [53]. An aliquot of LMWC derivatives (0.75 mL, 0.8–4.0 mg/mL for EG-LMWC and CM-LMWC; 0.1–0.8 mg/mL for AE-LMWC) or trolox (20–80 μg/mL) was added to a reaction mixture (2.25 mL) containing 840 μM NBT, 310 μM NADH, and 40 μM PMS in 0.1 M sodium phosphate buffer (pH 7.4). The absorbance was measured spectrophotometrically at 560 nm at 30-second intervals. The initial rate of increase in absorbance (A /min) was calculated. The capacity to scavenge superoxide radicals was calculated using the following equation:

$$\text{scavenging}\;\text{effect}\;\left(\%\right)=\left[1-{\left(\text{A}/\text{min}\right)}_{\text{sample}}/{\left(\text{A}/\text{min}\right)}_{\text{control}}\right]\times 100\%$$

### Measurement of oxygen radical absorbance capacity (ORAC)

ORAC assays were conducted as described by Ou et al. using a Synergy^™^ HT Multi-Mode Microplate Reader (BioTekInstruments, Inc.) [54]. To each well, 50 μL of fluorescein (78 nM) and 50 μL of sample (LMWC derivatives, 100 μg/mL), blank (H_2_O) or standard (trolox, 100 μM) were added. Twenty-five microliters of AAPH (221 mM) were then added. The reaction was performed at 37°C. The fluorescence was measured at 514 nm with an excitation wavelength of 490 nm. Measurements were taken every 5 min until the relative fluorescence intensity was less than 3% of the initial reading. The ORAC values, which were expressed as μmole trolox equivalents per gram of sample, were calculated using the following formula:

$$\text{ORAC}\;\left(\mu \text{mole}\;\text{trolox}\;/\;\text{g}\;\text{sample}\right)\;=\;\frac{AUC_{sample}-AUC_{blank}}{AUC_{trolox}-AUC_{blank}}\;\times \frac{\mu mole\;of\;trolox}{gram\;of\;sample}$$

where AUC is the area under the fluorescence decay curve. Micromole of trolox and gram of sample are the amount of trolox (μmole) or sample (gram) in the reaction mixture, respectively.

### Data analysis

Measurements were performed in triplicate when assaying the free radical-scavenging activities of LMWC derivatives. Other analytic measurements were performed in duplicate. Analysis of variance was performed using ANOVA with IBM SPSS Statistics 20. Mean values were compared by Fisher’s LSD (least significant difference) method. A significance level of less than 5% was adopted for all comparisons.

## Results

### Enzyme activities in jelly fig latex

As shown in Table 1, jelly fig latex contained a variety of exo-glycosidase activities in addition to protease, peroxidase and chitinolytic enzyme activities. Like most plants and plant latexes, jelly fig latex secreted a rich amount of proteases and peroxidases. The chitosanase activity was not remarkable as the protease or peroxidase activities. This was the first study to report on the characteristics of chitosanase in plant latex.

**Table 1:** Activities of proteases, chitnolytic enzymes, peroxidases and exo-glycosidases in the latex of jelly fig fruits^1^.

Enzyme	Substrate	Activity (unit /g)
**Protease**	Casein	85,163.4 **±** 1678^a^
**Chitinase**	Glycol chitin	58.6 ± 1.7^c^
**Chitosanase**	Chitosan	23.3 ± 0.7^c^
**Peroxidase**	*ο*-Phenylenediamine	17,823.1 ± 359^b^
**β-N-Acetylglucosaminidase**	*p*Np-β-GlcNAc	3.6 ± 0.2^x^
**α-Galactosidase**	*p*Np-α-Gal	3.6 ± 0.1^x^
**β-Galactosidase**	*p*Np-β-Gal	3.2 ± 0.1^x^^y^
**α-Glucosidase**	*p*Np-α-Glc	3.1 ± 0.2^x^^z^
**β-Glucosidase**	*p*Np-β-Glc	2.6 ± 0.4^y^^z^
**β-Xylosidase**	*p*Np-β-Xyl	2.7 ± 0.2^y^^z^
**β-L-Fucosidase**	*p*Np-β-L-Fuc	2.6 ± 0.2^y^^z^

^1^Data are presented as mean ± SD. (n = 2). Mean with different letters (a-c) within the same column differ significantly (*p* < 0.05).

^a-c^ values for endo-hydrolases and peroxidase in the same column with different superscripts show significant different (*p* < 0.05).

^x-z^ values for exo-glycosidases in the same column with different superscripts show significant different (*p* < 0.05).

### Chitosanase purification

**Fig 1:**
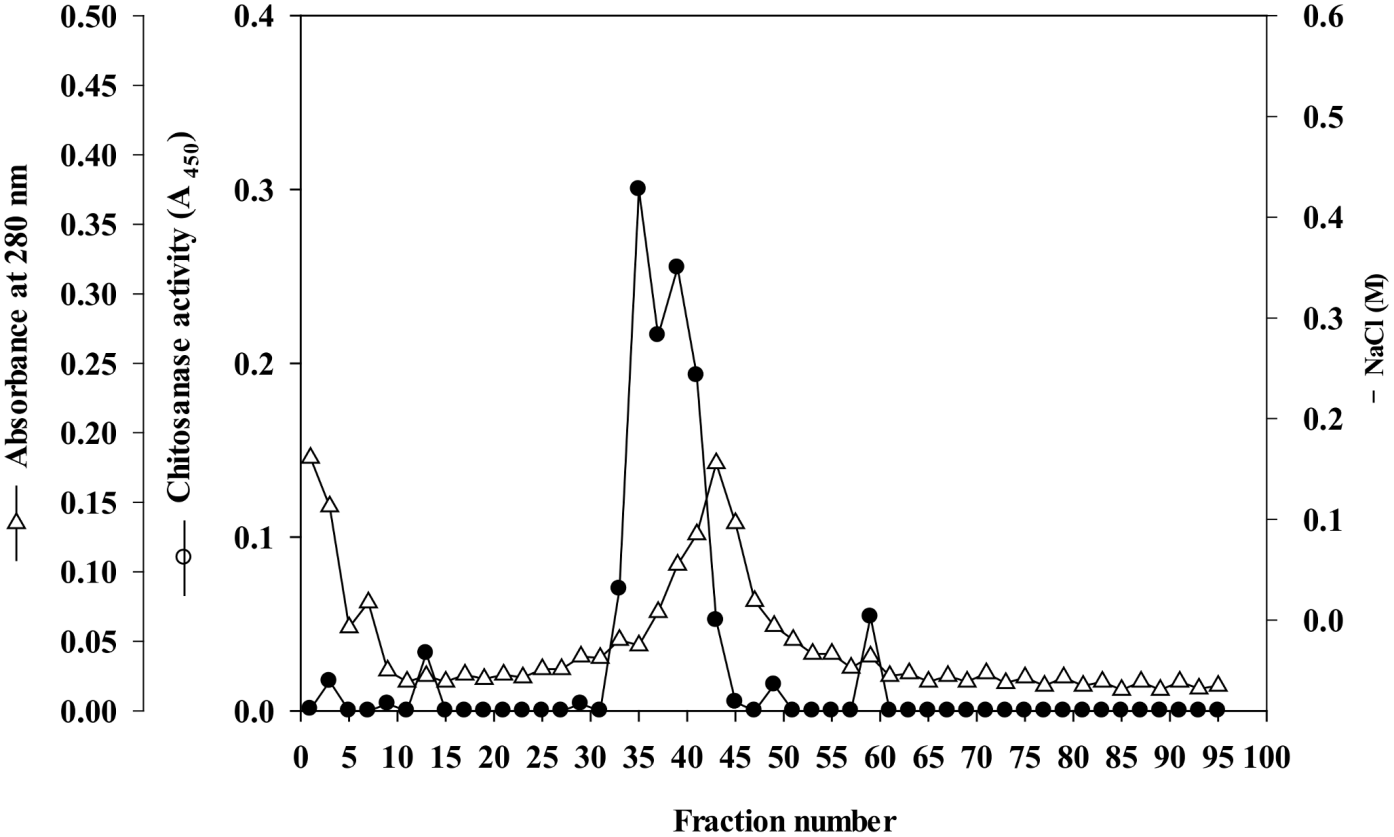
Ion-exchange chromatography of chitosanase on a DEAE-Sephacel column. The column (1.0 × 20 cm) was equilibrated with 0.025 M imidazole-HCl buffer (pH 7.4), after which the chitosanase obtained from the *p*APMA-Sepharose 4B column was applied. The bound proteins were eluted with a linear gradient of NaCl (0–0.5 M) in equilibrium buffer at a flow rate of 30 mL/h; 3 mL fractions were collected.

Chitosanase activity was present in the crude extract of jelly fig latex that precipitated in 50–80% saturated ammonium sulfate solution. Subsequently, a protein peak showing chitosanase activity was separated from the other proteins via gel filtration using Sephacryl S-100 HR column. The protein was further purified by affinity chromatography on a *p*APMA-Sepharose column. The chitosanase was not adsorbed onto the gel and emerged immediately from the column. Finally, the chitosanase was eluted with 0.15–0.20 M NaCl in imidazole-HCl buffer by ion-exchange chromatography on a DEAE-Sephacel column (Fig 1). As shown in Table 2, the purity of the enzyme was estimated to be approximately 7.6-fold greater than that of the ammonium sulfate precipitate. The final yield was 9.5%.

**Table 2:** Purification of chitosanase from jelly fig latex^1^.

Procedure	Total volume (mL)	Total acativity (mU)^2^	Total protein (mg)	Specific activity (mU/ mg)	Purification (fold)	Yield (%)
**(NH**_**4**_**)**_**2**_ **SO**_**4**_ **(50–80% satruation)**	5	13177	80	165	1.0	100
**Sephacryl S-100 HR gel filtration**	90	5017	25	201	1.2	38
***p*APMA-Sepharose adsorption**	14	2247	40	562	3.4	17
**DEAE-Sephacel ion-exchange chromatography**	26	1248	1.0	1248	7.6	9.5

^1^Data were obtained from 0.5 g of lyophilized jelly fig latex.

^2^Chitosanase activity was determined using aminoethyl chitosan as the substrate. One mU is 10^−3^ units.

### Molecular mass

**Fig 2:**
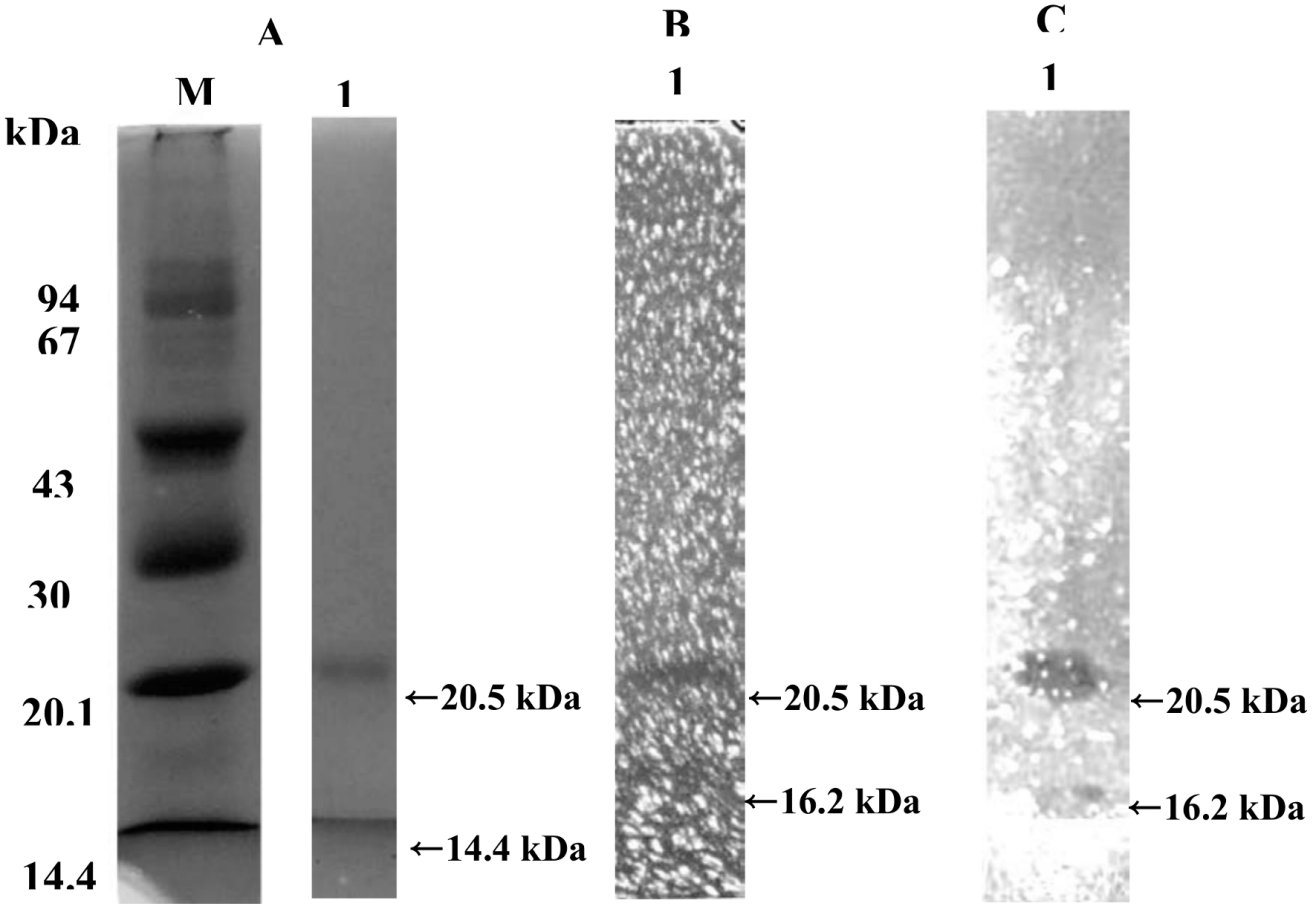
SDS-PAGE of the purified chitosanase. Electrophoresis was performed in 12.5% acrylamide gel containing glycol chitosan (B) or glycol chitin (C). Chitosanase or chitinase activity was detected by Calcofluor White M2R staining after the lysis of glycol chitosan or glycol chitin in the gel. Lane M contains the protein molecular weight marker; lane 1 contains purified chitosanase.

There were two protein bands present in the SDS-PAGE, with molecular masses of 20.5 kDa and 14.4 kDa (Fig 2A). Chitinolytic activity staining analysis confirmed that the protein with a molecular mass of 20.5 kDa was a chitosanolytic enzyme exhibiting both chitosanase activity (Fig 2B) and chitinase activity (Fig 2C). However, the protein with molecular mass of 14.4 kDa showed neither chitosanase activity nor chitinase activity. Both gels stained for chitosanase (Fig 2B) and chitinase (Fig 2C) activities showed trace band of activity at a molecular mass of 16.2 kDa. These results indicated that the purified enzyme contained at least three proteins (20.5 kDa, 16.2 kDa and 14.4 kDa). Two of these proteins, those with the molecular masses of 20.5 kDa and 16.2 kDa, were chitinolytic enzymes with dual chitinase and chitosanase activities. However, the protein with a molecular mass of 16.2 kDa was not abundant enough to be detected in a protein-stained SDS-PAGE gel.

### Isoelectric point (p*I*)

**Fig 3:**
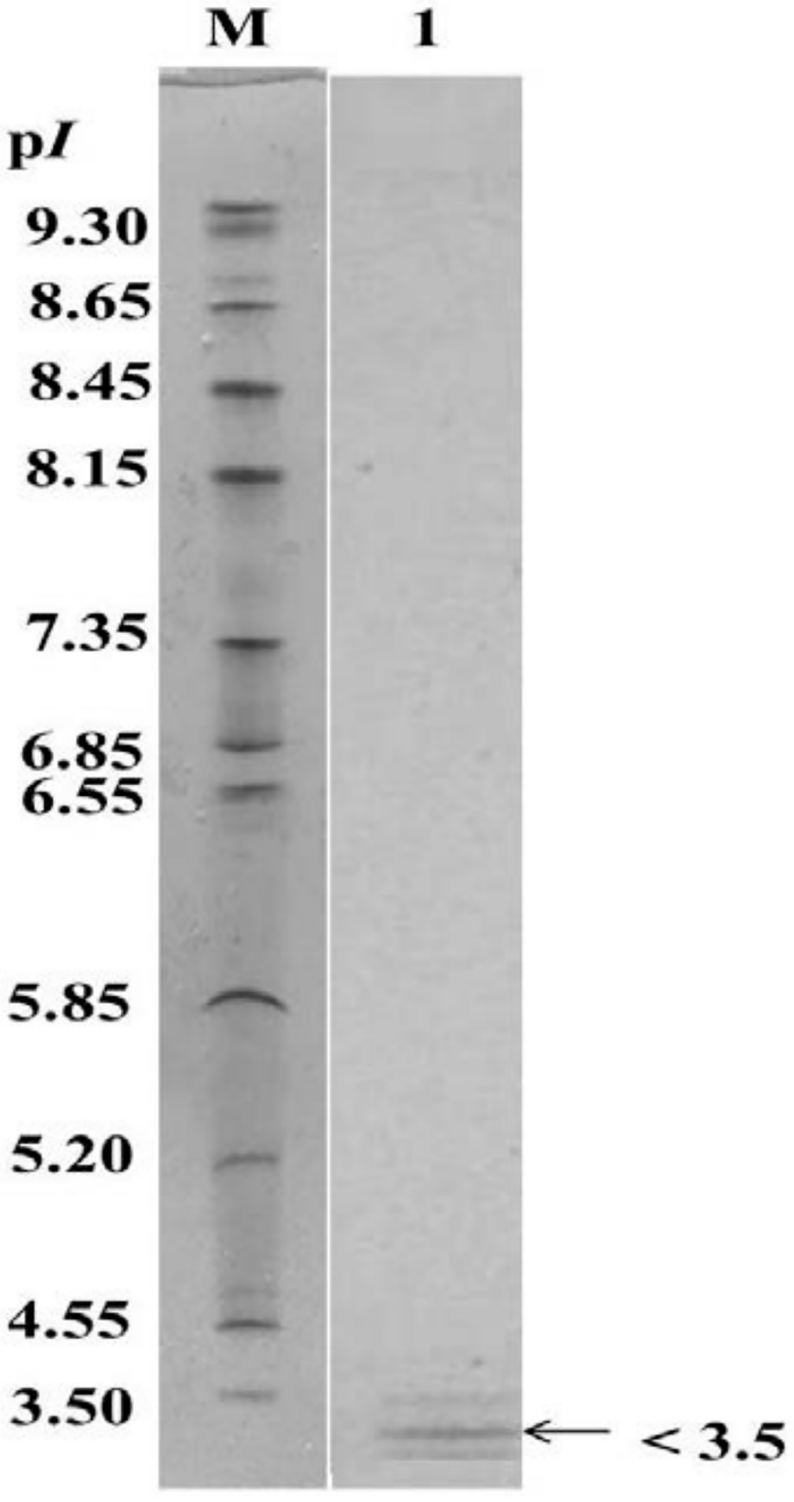
IEF-PAGE of the purified chitosanase. IEF-PAGE was performed on a PhastGel IEF 3–9 gel containing wide-range ampholytes (p*I* 3–10). Lane M contains p*I* marker proteins; lane 1 contains purified chitosanase. Proteins were detected by Coomassie Blue R-250 staining.

The p*I* of the purified enzyme was < 3.5, as analyzed by IEF electrophoresis and protein staining (Fig 3). This result indicated that the purified enzyme was an acidic chitosanase.

### Effects of pH and temperature on enzyme activity

The optimal pH and temperature for chitosan hydrolysis by the purified chitosanase were 4.5 and 50°C, respectively (data not shown).

### Effect of chitosan deacetylation on enzyme activity

As shown in Table 3, chitosan polymers with various degrees of deacetylation (21–94%) were all susceptible to purified chitosanase. Hydrolysis was most effective on 70% deacetylated chitosan; 94% deacetylated chitosan was the least susceptible.

**Table 3:** Effect of the degree of chitosan deacetylation on the activity of the purified chitosanse^1^.

Degree of chitosan deacetylation (%)	Relative chitosanase activity (%)^2^
21	70.3 ± 1.2^df^
31	79.4 ± 6.9^bd^
40	82.0 ± 2.1^bc^
52	85.2 ± 2.8^b^
64	68.7 ± 1.7^ef^
70	100.0 ± 2.5^a^
84	75.2 ± 2.2^cde^
94	54.2 ±1.6^g^

^1^Data are presented as mean ± SD. (n = 2). Mean with different letters (a-g) within the same column differ significantly (*p* < 0.05).

^2^ The relative activity was expressed as the percentage ratio of the enzyme with tested substrate to that with 70% deacetylated chitosan.

### Substrate specificity

The purified chitosanase hydrolyzed chitin, chitosan and their derivatives are shown in Table 4. When a value of 100 was arbitrarily assigned for the activity of purified enzyme toward chitosan, the enzyme activities towards glycol chitin, aminoethyl chitosan, glycol chitosan, carboxymethylchitosan and colloidal chitin were 96.2 ± 2.5, 80.2 ± 8.3, 66.1 ±2.0, 65.1 ± 9.7 and 23.0 ± 0.9, respectively. These results were consistent with the gel activity staining findings for chitinase and chitosanase in SDS-PAGE (Fig 2B and 2C).

**Table 4:** Substrate specificity of the purified chitosanase^1^.

Substrate	Relative activity (%)^2^
Chitosan	100.1 ± 2.1^a^
Glycol chitin	96.2 ± 2.5^a^
Aminoethyl chiosan	80.2 ± 8.3^b^
Glycol chitosan	66.1 ±2.0^bc^
Carboxymethyl chitosan	65.1 ± 9.7^c^
Colloidal chitin	23.0 ± 0.9^d^

^1^Data are presented as mean ± SD. (n = 2). Mean with different letters (a-d) within the same column differ significantly (*p* < 0.05).

^2^ The relative activity was expressed as the percentage ratio of the enzyme with tested substrate to that with chitosan.

### Maximal velocity and Michaelis constant

The maximal velocity (*V*_max_) and Michaelis constant (*K*_m_) of the purified chitosanase for chitosan hydrolysis was 0.69 μmole min^-1^ mg^-1^ and 0.089 mg mL^-1^, respectively (data not shown).

### Products of the hydrolysis of chitosan derivatives

**Fig 4:**
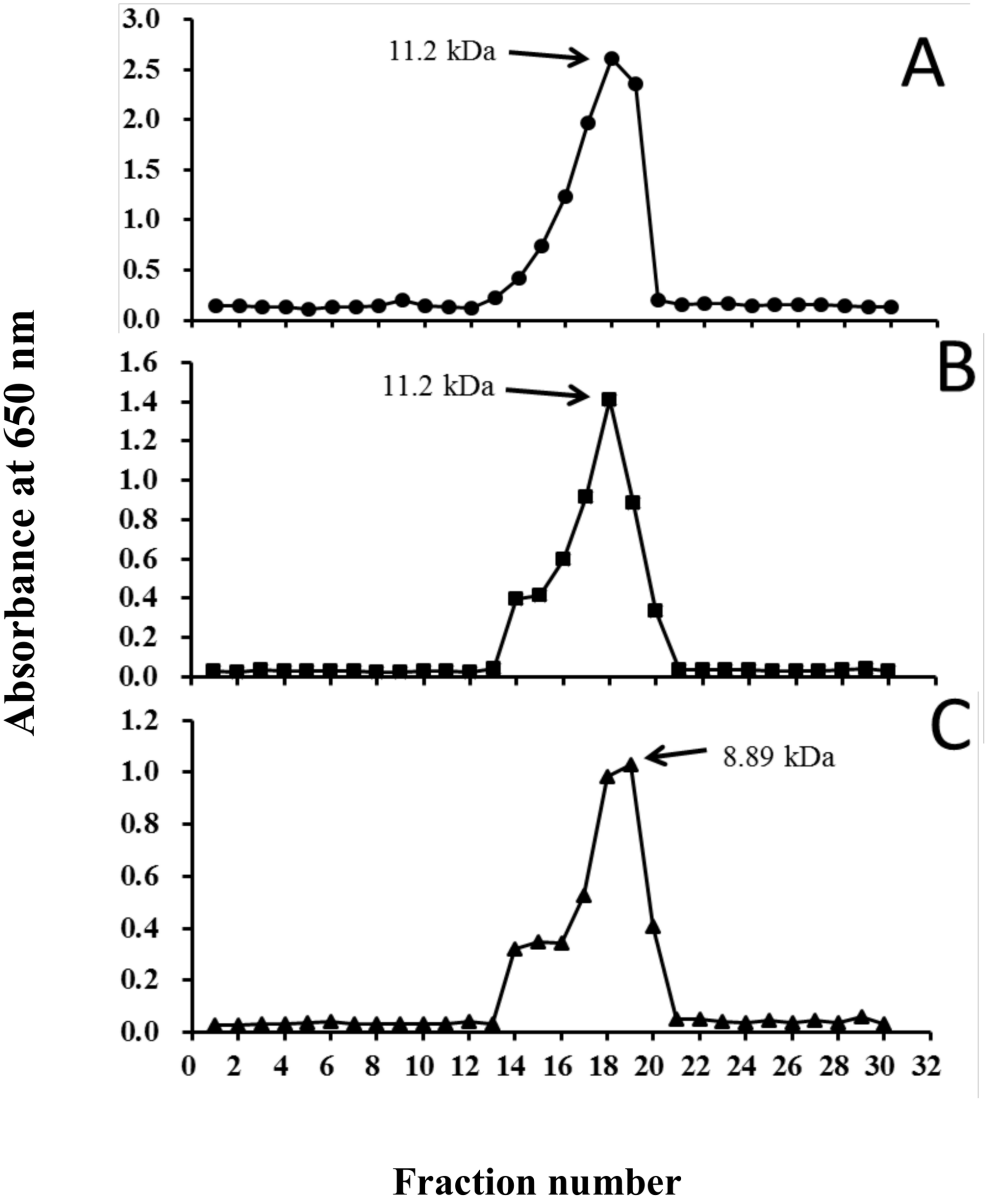
Gel filtration of LMWC derivatives on a Superose 12 HR column: (A) EG-LMWC; (B) CM-LMWC; (C) AE-LMWC.

The water-soluble chitosan derivatives EG-chitosan, CM-chitosan and AE-chitosan were digested with a water extract of jelly fig latex. After digestion, the depolymerized chitosan hydrolysates from EG-chitosan, CM-chitosan and AE-chitosan were named EG-LMWC, CM-LMWC and AE-LMWC, respectively. Gel filtration showed that all of the hydrolysates were fractionated into a single peak. The average molecular weights of EG-LMWC, CM-LMWC and AE-LMWC were 11.2, 11.2 and 8.89 kDa, respectively (Fig 4).

### Free radical scavenging activities of the LMWC derivatives

ABTS, superoxide and ORAC free radical scavenging assays were used to measure the antioxidant activities of EG-LMWC, CM-LMWC, AE-LMWC and COS. As shown in Table 5, all of the LMWC derivatives showed scavenging activities toward the three tested free radicals, with different antioxidant capacities.

**Table 5:** Free radical-scavenging activities of LMWC derivatives^1^.

LMWC derivatives	TEAC (mg trolox/ g sample)		ORAC (*u*mol trolox/ g sample)
	ABTS radical	Superoxide radical	Peroxyl radical
EG-LMWC	29.0±0.3^c^	16.8±1.3^b^	1561±206^b^
CM-LMWC	46.0±0.1^a^	18.5±0.6^b^	1894±347^ab^
AE-LMWC	40.1±0.3^b^	1853±52^a^	2175±11^a^
COS	7.4±0.2^d^	51.5±0.0^b^	1140±42^c^

^1^ Data are presented as mean ± SD. (n = 3). Mean with different letters within the same column differ significantly (*p* < 0.05).

LMWC, low-molecular-weight chitosan; EG, ethylene glycol; AE, aminoethyl; CM, carboxymethyl; COS, commercial chitooligosaccharides (MW < 5 kDa).

The ABTS radical scavenging assay detects the inhibitory effect of a sample on ABTS^․+^ cation radicals. The trolox equivalent antioxidant capacity (TEAC) values for EG-LMWC, CM-LMWC, AE-LMWC and COS toward ABTS^․+^ radicals were 29.0±0.3, 46.0±0.1, 40.1±0.3 and 7.4±0.2 mg trolox/g sample, respectively. Towards ABTS^․+^ radicals, CM-LMWC had the highest scavenging activity, followed by AE-LMWC, EG-LMWC and COS, respectively.

Superoxide anion radicals (^•^O_2_^-^) are the one-electron reduced form of molecular oxygen. The superoxide radical assay is based on the capacity of a sample to inhibit superoxide radicals. The TEAC values for EG-LMWC, CM-LMWC, AE-LMWC and COS toward superoxide radicals were 16.8±1.3, 18.5±0.6, 1853±52 and 51.5±0.0 mg trolox/g sample, respectively. AE-LMWC had the highest superoxide radical-scavenging capacity and was much more effective than the other two LMWC derivatives.

**Fig 5:**
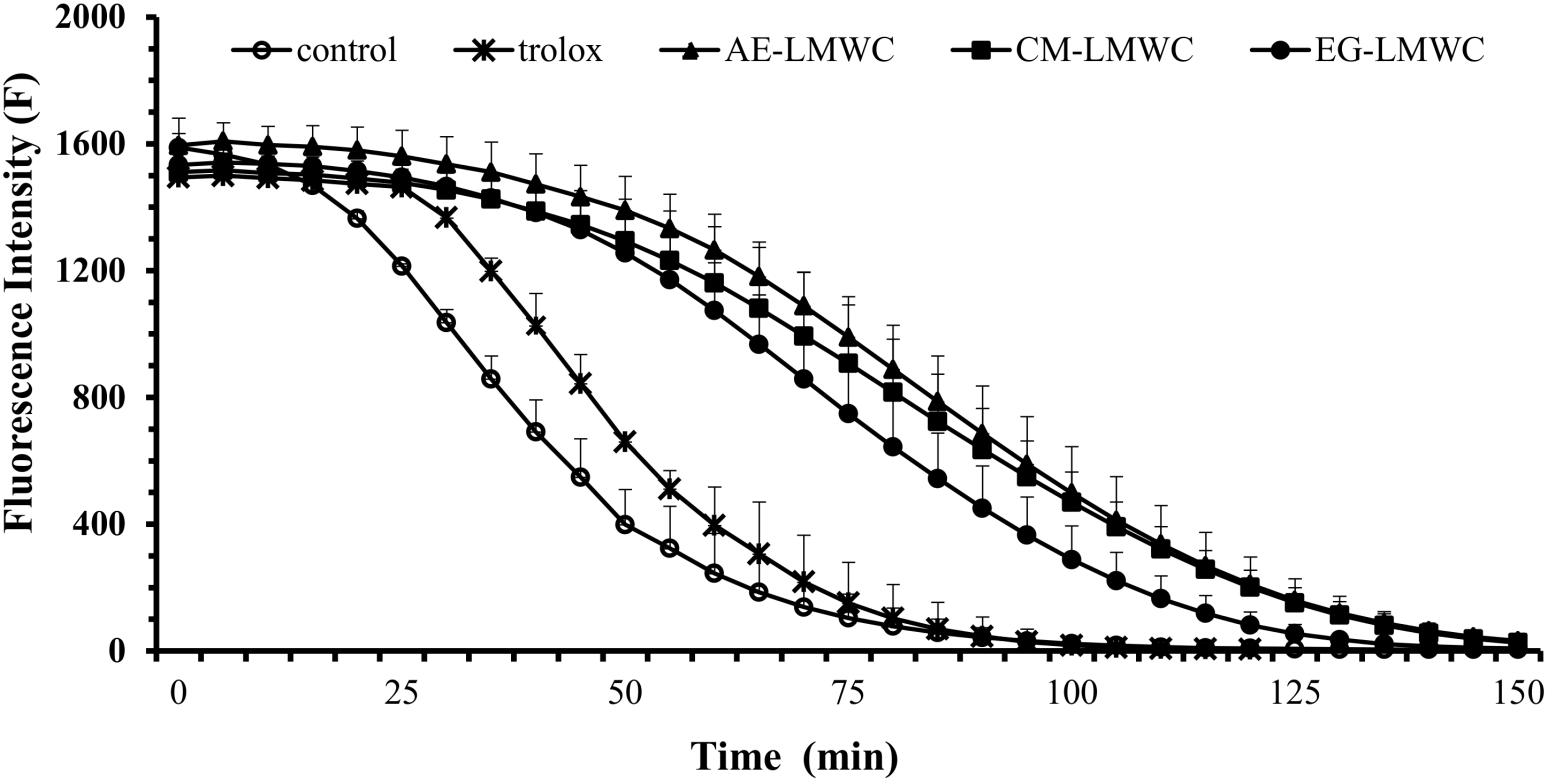
Radical scavenging activities of LMWC derivatives and trolox toward peroxyl radicals. Each value is the mean ±SD. (n = 3).

The ORAC assay was used to test the antioxidant capacities of the LMWC derivatives to quench peroxyl radicals (ROO^․^). Fluorescence decay curves between the blank and the LMWC derivatives are shown in Fig 5. Trolox was used as a positive control. The ORAC values for EG-LMWC, CM-LMWC, AE-LMWC and COS were 1561±206, 1894±347, 2175±11 and 1140±42 μmol trolox/g sample, respectively. All of the LMWC derivatives exhibited significant peroxyl radical-scavenging capacities. Moreover, the scavenging capacities of AE-LMWC and CM-LMWC were superior to that of EG-LMWC and COS.

To sum up the above antioxidant studies, all of the LMWC derivatives showed significant antiradical capacities. Scavenging capacities were particularly great against peroxyl radicals.

## Discussion

Many plants secret latex from wounds caused by the invasion of predators such as insects and microorganisms. Plant latexes are rich sources of many types of enzymes and proteins. In plant latex, proteases, exo-glycosidases, chitinolytic enzymes and peroxidases may serve in the defense against invasion. The presence of exo-glycosidases in plant latex may also be involved in the cell-wall degradation process during the differentiation of articulated laticifers [55]. Proteases in latex could digest the peritrophic membrane of the insect midgut [56]. Because chitin is a major component of various insect tissues and a major constituent of cell walls in fungi, chitinases in plant latex play defensive roles against fungi and insects [18].

In the present study, we isolated and characterized a chitosanolytic enzyme from the soluble fraction of jelly fig latex. We also found that a substantial amount of proteases and peroxidases, as well as a variety of exo-glycosidases, were present in jelly fig latex.

The purified chitosanase had a molecular mass of 20.5 kDa and dual chitinase and chitosanase activities, as determined by SDS-PAGE and gel activity staining. This enzyme is different from the chitinase (30 kDa) previously isolated from jelly fig achenes in molecular mass and substrate specificity [37, 57]. Like other proteins present in jelly fig latex, the enzyme isolated here may play a crucial role in the defense against predators such as insects and microorganisms.

Chitinases are widely found in plant latex [18,23–27]. At present, little information has been reported concerning plant chitosanases; in particular, few reports exist concerning chitosanases from plant latex. In our previous work, a chitosanase (16.6 kDa) was isolated from a commercial ficin preparation. This enzyme exhibited both chitosanase activity and chitinase activity [45]. Kitajim et al. [27] purified two chitinase-like proteins (46–50 kDa) with dual chitinase and chitosanase activities from latex of mulberry. In the present study, the purified chitosanase (20.5 kDa) also showed dual chitinase and chitosanase activities. Plant chitinases or chitosanases with dual chitinase and chitosanase activities are not rare. Plant chitosanases are distinguished from chitinases (EC 3.2.1.14) by their lower apparent molecular mass and their substrate specificity [58]. Chitosanases and chitinases may not be conclusively distinguished. Usually, chitinases and chitosanases work together to degrade the cell wall of pathogenic fungi present in plant wounds. This may be why chitinases and chitosanases isolated from plants frequently exhibit dual chitinase and chitosanase activities.

Recently, Raskovic et al. (2015) measured changes in enzyme activities in *Ficus carica* latex during fruit ripening. These findings suggested that shifts occur in the peak activities of each defense-related enzyme during fruit ripening, which are serial strategies against insects and fungi [59]. Chitinolytic activity in *Ficus carica* latex at the beginning of flowering was 6.5 times greater than that when the fruit was ripe. In this study, jelly fig latex was collected during the ripening period of fruits; the yield of purified chitosanase was 9.5%. We expect that the yield of the chitosanase will be higher if we collect the latex during peak chitosanase activity.

The purified chitosanase showed substantial chitosan degradation with various degrees (21–94%) of deacetylation. These findings indicate that purified chitosanase can be used on a wide range of deacetylated chitosans.

The antioxidant capacity of chitosan serves a broad range of food applications, such as preventing lipid oxidation and preserving meat products [60–63]. Low molecular weight chitosans (LMWCs) exhibited greater antioxidant activity than high molecular weight chitosans. Thus, more and more LMWCs are used as antioxidant agents. For example, LMWCs are utilized in the preservation of crab and Kamaboko gels and the preparation of edible antioxidant film [64–67]. In this study, the hydrolysate products of chitosan derivatives, named EG-LMWC, CM-LMWC and AE-LMWC, exhibited significant free radical scavenging activities, and the free radical scavenging activities of those chitosan derivatives were greater than commercial COS. CM-LMWC and AE-LMWC showed superior activity towards ABTS^․+^ and peroxyl free radicals compared to EG-LMWC. AE-LMWC was more effective towards superoxide radicals than the other two LMWC products. The difference in scavenging capacities against various free radicals may be due to the combined effects of differences in the electron cloud density of the substituting groups and the accessibility towards various free radicals. The above results suggest that it is feasible to apply the purified chitosanase to the production of LMWCs with antioxidant activity.

## Conclusion

In the present study, we isolated and characterized a chitosanase from jelly fig latex. The chitosanase identified is a bifunctional enzyme with dual chitinase and chitosanase activities. The enzyme catalyzed the hydrolysis of chitin and chitosan polymers and their derivatives. This is a valuable enzyme that can be used for the production of water-soluble low molecular weight chitosans.
